# Identifying causes and associated factors of stillbirths using autopsy of the fetus and placenta

**DOI:** 10.1007/s00404-024-07522-1

**Published:** 2024-05-01

**Authors:** Eliel Kedar Sade, Daniel Lantsberg, Moriel Tagar Sar-el, Sheizaf Gefen, Michal Gafner, Eldad Katorza

**Affiliations:** 1 Department of Obstetrics and Gynecology, The Edith Wolfson Medical Center, Holon, Affiliated With the Faculty of Medical and Health Sciences, Tel-Aviv University, Tel Aviv, Israel; 2 Department of Obstetrics and Gynecology, The Royal Women’s Hospital, Melbourne, Australia; 3 Department of Dermatology, Sheba Medical Center, Ramat-Gan, Israel; 4 Department of Internal Medicine ‘E’, Tel Aviv Medical Center, Tel Aviv, Israel; 5 Department of Pediatrics B, Schneider Children’s Medical Center of Israel, Petach Tikva, Israel; 6 Faculty of Medical and Health Sciences, Tel-Aviv University, Tel Aviv, Israel; 7 Gertner Institute for Epidemiology & Health Policy Research, Sheba Medical Center, Ramat-Gan, Israel; 8 Department of Obstetrics and Gynecology, Sheba Medical Center, Ramat-Gan, Israel

**Keywords:** Fetal death, Fetal demise, Post-mortem, Pathology

## Abstract

**Purpose:**

The study aimed to evaluate the causes of death and associated factors in cases of stillbirth, using post-mortem examination and applying a rigorous, evidence-based holistic approach.

**Methods:**

Our retrospective observational study included cases of autopsy following stillbirth that occurred at our tertiary medical center during a period of 8 years. Detailed up-to-date criteria that incorporate clinical reports, medical history, prenatal imaging, and histopathological findings were used to evaluate the cause of death and associated factors.

**Results:**

After applying our proposed methodology, 138 cases of stillbirth were classified into eight categories based on the causes of death. A definitive cause of death was observed in 100 (72%) cases, while 38 (28%) cases were considered unexplained. The leading cause of death was placental lesions (n = 39, 28%) with maternal vascular malperfusion (MVM) lesions being the most common (54%). Ascending infection was the second most common cause of fetal death (n = 24, 17%) and was often seen in the setting of preterm labor and cervical insufficiency.

**Conclusion:**

The largest category of cause of death was attributed to placental pathology. Using rigorous detailed up-to-date criteria that incorporate pathological and clinical factors may help in objectively classifying the cause of death.

**Supplementary Information:**

The online version contains supplementary material available at 10.1007/s00404-024-07522-1.

## What does this study add to the clinical work

**Table:** 

The study highlights the significance of fetal and placental autopsy for determining the cause of fetal death. It also introduces stringent criteria to objectively classify the cause of death, potentially reducing cases of unexplained fetal demise.	

## Introduction

Stillbirth is a devastating obstetric complication that remains a major, largely unstudied, cause of perinatal death, affecting 1 in 175 deliveries in the United States, with approximately 2.6 million deaths a year worldwide [1–3]. Unfortunately, a history of stillbirth increases the risk of subsequent stillbirth by almost five-fold and causes significant anxiety in future pregnancies, especially in cases that remain unexplained [4]. Thus, it is of the utmost importance to establish an accurate cause of fetal death to allow a better estimate of recurrence risk and adequate counseling in future pregnancies.

In recent years, our knowledge of stillbirth has advanced tremendously thanks to post-mortem examinations of the placenta, with a variety of classification systems developed to identify the cause of fetal death. Nevertheless, our current understanding of the causes of stillbirth remains limited due to a lack of consensus terminology, different approaches, and subjective utilization of numerous classification systems. This is strongly reflected by the wide variation in the proportion of stillbirths classified as 'unexplained' in the literature (9–71%) [5–9]. Additionally, the field of placental pathology is changing rapidly, with recent studies identifying multiple placental lesions in apparently normal pregnancies, thus undermining our current understanding of the role of placental lesions in the development of stillbirth [10, 11]. It has been suggested, that the use of a more holistic approach, which embodies pathological findings, clinical reports, medical history and prenatal imaging can be more effective in drawing conclusive conclusions and ascertaining the cause of death [6, 7].

The aim of our study was to evaluate cases of stillbirths in our tertiary hospital, using rigorous evidence-based criteria, to identify definitive causes and associated characteristics of stillbirths.

## Methods

This retrospective observational study included details of fetal autopsies following stillbirth performed between 2006 to 2013 at our tertiary medical center. Following parental informed consent for both the post-mortem examination and the study, specialized pathologists at our Pathological Institute, each possessing expertise in fetopathology, conducted a complete conventional autopsy encompassing gross, histological, and bacteriological evaluations of the fetus, placenta, membranes, and umbilical cord. These autopsy reports were retrospectively revised using the Amsterdam Placental Workshop Group international criteria to align with current diagnostic standards. Inclusion criteria were fully detailed autopsy reports of singleton and twin pregnancies following early or late IUFD and stillbirth. Complementary information, retrospectively recorded in our database, was utilized for a complete evaluation to support and strengthen the determination of the cause of stillbirth in our study. These included a thorough clinical evaluation of maternal medical and obstetric history, previous stillbirths, and relevant clinical conditions. Prenatal data, including maternal blood tests, along with comprehensive serologic analysis (HIV, syphilis, cytomegalovirus, toxoplasmosis, rubella virus, parvovirus, HBV, and HCV), antenatal biochemical screening and ultrasound scans. Additional prenatal evaluations, such as fetal and umbilical Doppler ultrasound, fetal echocardiography, fetal magnetic resonance imaging (MRI) scans, and genetic tests (karyotyping or microarray), preformed per clinical indication, were also recorded in our database when available.

The primary outcome of our study was to determine the cause of death in each case using autopsy reports and complementary data as described above. For the purpose of the study, all cases were classified based on gestational age at autopsy as follows: extremely preterm (< 28 weeks), early preterm (28–34 weeks), late preterm (34–37 weeks) and term (> 37 weeks) IUFDs. After reviewing autopsy reports and complementary data, a definitive cause of death was assigned to each case. *Placental abruption* was considered the cause of death in cases with a clinical evidence of placental abruption (such as sonographic diagnosis or the presence of retroplacental clots or bleeding). *Placental lesions*, specifically those of high burden displaying diffused and high-grade severity, were identified as the predominant contributors leading to the fatal outcome, following classification according to the Amsterdam guidelines. This included high burden lesions (defined as two or more lesions) of maternal vascular malperfusion (MVM) or fetal vascular malperfusion (FVM), inflammatory lesions (high grade and extensive), and specific stillbirth-associated lesions such as massive perivillous fibrin deposition (MPVFD). Lesions of delayed villous maturation (DVM) were regarded as causative only in the presence of severe features and when the placenta was devoid of other pathologies) [11]. The full classification of the histopathological lesions is depicted in detail in Table S1. To enable further evaluation of placenta-origin clinical conditions, in cases with a known predominant clinical condition that exhibited severe pathological features, the cause of death was classified based on the underlying clinical condition [12]. These included cases of clinically reported preeclampsia with high burden maternal vascular malperfusion (MVM) lesions and clinically recorded placental abruption with accompanying pathological features of retroplacental hemorrhage. Cases with only mild features of placental lesion (such as small fraction of retroplacental hematoma or focal fibrin depositions) were excluded from this category. *Ascending infection* was regarded as cause of death in the presence of histopathologic evidence of high stage maternal inflammatory response (MIR, stage 3) or any stage of fetal inflammatory response (FIR). Cases with only isolated low stage MIR (stages 1 and 2), which can also be seen in placentas of normal pregnancies, were considered insignificant [10, 13, 14]. *Non-placental Intrauterine growth restriction* (IUGR) was defined as the cause of death in cases of prenatally estimated fetal weight below the 10th percentile with no evident of placental-related pathology [15]. Cases of IUGR in which a substantial placental-related pathology was evident (such as maternal vascular malperfusion lesions), were categorized as placental cause of death. To allow distinction between abnormal IUGR and normal SGA, cases with birth weight below the 10th percentile with a normal prenatal ultrasound and no additional evidence of additional pathological etiologies, including infections, congenital malformations, or chromosomal abnormalities were classified as normal SGA. *Cord abnormality* included cases of cord entrapment, true knot or stricture and compromised fetal microcirculation. As the clinical significance of these abnormalities has yet to be proven [16, 17], only cases with a confirmed histopathologic evidence of umbilical cord abnormality (such as umbilical blood flow restriction) were regarded as cause of death [18, 19]. *Fetal abnormality* was the presence of a fetal condition, identified at autopsy, that markedly increased the risk of fetal death. This category included severe fetal malformations (such as bilateral renal agenesis and severe CNS malformations), lethal fetal conditions (e.g., erythroblastosis fetalis and neonatal hemochromatosis), and fatal genetic abnormalities (such as trisomy 18). Cases in which a known infection-induced fetal injury was detected (such as pneumonia or intraventricular hemorrhage secondary to ascending infection) were classified as ascending infection [20, 21]. Minor malformations that are compatible with life (such as facial or digital deformities) were classified as insignificant. In the absence of a definitive cause of death (based on clinical, histopathological, and imaging findings) the case was classified as *unexplained*.

For the purpose of the study, potential characteristics and conditions that may increase the risk of stillbirth but are not considered causal, rather associated with stillbirth, were further analyzed. These non-lethal stillbirth-association factors were compared between the different causative factors to allow further investigation in this area. These included maternal medical history (diabetes mellitus, hypertension, hypercoagulability and hypothyroidism) and abnormal obstetric history (history of two or more spontaneous abortions, stillbirth or IUFD); obstetric complication such as premature rupture of membrane (PROM), cervical insufficiency and preterm delivery; amniotic fluid abnormalities (polyhydramnios or oligohydramnios detected in prenatal ultrasound); small-for-gestational-age (SGA) neonates; mild congenital malformations that do not pose immediate risk for the fetus; and histopathological lesions not sufficiently severe to result in stillbirth, such as mild features of MVM or low-grade ascending infection, cord abnormalities with no evidence of compromised microcirculation (such as cord insertion, marginal or velamentous), abnormal coiling index, and single umbilical artery.

The study protocol has gained the approval of the regional ethical committee, and all patients gave informed consent.

This is a retrospective observational study; frequencies of occurrence are described as percentages. Statistical analyses were performed using SPSS software (IBM SPSS Statistics for Windows, ver. 23, IBM corp., Armonk, NY, USA, 2015) and NCSS software (NCSS 2021, Kaysville, Utah, USA, 2021) were used for. Continuous variables were evaluated for normal distribution and reported as mean (standard deviation) and median (interquartile range).

## Results

During a period of 8 years, a total of 265 cases of autopsies following fetal death were documented in our database. After excluding 115 cases of elective termination of pregnancy and 12 cases with insufficient data, 138 cases of autopsies preformed after stillbirths were recorded in our database. Table 1 depicts the clinical characteristics of the study group. The mean maternal age was 32.3 (standard deviation [SD], 5.3 years). The majority of the women were multigravida (n = 90, 65%), while almost half were nulliparous (n = 61, 44%). The mean gestational age at the time of stillbirth was 28.6 weeks (SD 7.6 weeks). There were 69 extremely preterm (< 28 weeks), 22 early preterm (28–34 weeks), 18 late preterm (34–37 weeks) and 29 term (> 37 weeks) IUFDs. Of all the cases, 116 (84.1%) were singletons, and the remaining 22 (16%) were twins. The majority of stillbirths were appropriate for gestational age (AGA, n = 94, 68%) with a median gestational weight at autopsy of 818 g (range 322–2415). Out of 37 small for gestational age (SGA) fetuses, only 14 (38%) were prenatally diagnosed with growth restriction. Relevant maternal medical conditions included 22 (16%) cases with an underlying thrombophilic conditions (history of DVT, genetic mutations such as MTHFR, FVL and protein S deficiency, APLA syndrome), 9 (7%) hypertensive disorders (chronic hypertension, gestational hypertension, or preeclampsia). Diabetes (diabetes mellitus or gestational diabetes) and hypothyroidism were found in 10 (7%) cases, respectively.

**Table 1:** Clinical characteristics of the study population

Total (N = 138)	Value
Maternal characteristics	
Maternal age (years)	32.3 ± 5.3*
Parity	
0	61 (44%)
1	41 (30%)
2	20 (14%)
3 +	16 (12%)
Gravidity	
1	48 (35%)
2–4	71 (51%)
5 +	19 (14%)
Number of spontaneous abortions	
0	103 (75%)
1 +	35 (25%)
Hypertension	9 (7%)
Diabetes	10 (7%)
Thrombophilia	22 (16%)
Hypothyroidism	10 (7%)

Fetal characteristics	
Male Sex	72 (54%)
Type of pregnancy	
Singleton	116 (84%)
Twin	22 (16%)
GA at autopsy (weeks)	28.6 ± 7.6*
Term birth	29 (21%)
Late preterm	20 (14%)
Early preterm	20 (14%)
Extremely preterm	69 (50%)
Fetal weight at autopsy (g)	818 (322–2415)^†^
AGA at autopsy	94 (68%)
LGA at autopsy	7 (5%)
SGA at autopsy	37 (27%)
Known IUGR	16 (12%)

Term (> 37 weeks), late preterm (34 to 37 weeks), early preterm (28 to 34 weeks), extremely preterm (< 28 weeks)

*GA* gestational age, *AGA* appropriate for gestational age, *LGA* large for gestational age, *SGA* small for gestational age, *IUGR* intrauterine growth restriction

^*^Mean ± standard deviation

^†^Median (interquartile range)

The distribution of the causes of stillbirth is presented in Table 2. In our cohort, a definitive cause of death was observed in 100 (72%) cases, while 38 (28%) cases were considered unexplained. Overall, the leading cause of death was placental lesions (n = 39,28%) with maternal vascular malperfusion (MVM) lesions being the most common, accounting for 21 cases with MVM lesions severe enough to result in fetal death. These were followed by fetal vascular malperfusion lesions (FVM, n = 7, 18%), inflammatory lesions (n = 4, 10%), severe delayed villous maturation (DVM, n = 4, 10%) and massive perivillous fibrin deposition (MPVFD, n = 3, 8%). The full classification of these cases is presented in Table 3. Cases with MVM lesions were mostly extremely and early preterm (43% and 38%, respectively, mean GA = 28.7), while FVM lesions were mostly late preterm and term (43% and 29%, respectively, mean GA = 33.5). Ascending infection was the second most common cause of fetal death, accounting for 24 (17%) cases. Chorioamnionitis tended to occur earlier in pregnancy (extremely preterm, 75%) and had the lowest mean GA of 24.6 weeks (SD 7.6). Additional 16 cases with milder features of chorioamnionitis (such as focal subchorionitis or cases without fetal response) were excluded from this category. Cord abnormality was the cause of death in 12 (9%) cases, and all were consistent with a histopathological features of blood flow restriction (such as compressed or congestive cord segments and obstructing thrombus in umbilical vessels). These cases tended to occur later in pregnancy and had a higher mean of GA (32.0 weeks, SD 7.6). Fetal abnormalities resulting in stillbirth were recorded in 8 cases, these included 2 cases of bilateral renal dysplasia and 1 case of each of the following: congenital heart malformation with severe lung hypoplasia, extensive hemorrhagic pulmonary embolism, neonatal hemochromatosis, erythroblastosis fetalis, anomaly consistent with trisomy 18, and mediastinal germ cell tumor compressing systemic veins. After reviewing clinical data and histopathological reports, 28% of the cases were regarded as unexplained, with no significant pathology that could be regarded as causative. These cases were characterized by a mean GA of 29.1 (SD 7.6 weeks) and were more frequently preterm (77%) and less frequently associated with term birth (23%).

**Table 2:** Classification of all cases based on the cause of death and with accordance to gestational age at autopsy

Cause of death	Value	GA (mean ± SD)	Extremely preterm	Early preterm	Late preterm	Term
Placental lesions	39 (28)	29.8 ± 7.0	16 (41)	9 (23)	7 (18)	7 (18)
Ascending infection	24 (17)	24.6 ± 7.6	18 (75)	1 (4)	2 (8)	3 (13)
Cord abnormality	12 (9)	32.0 ± 7.6	3 (25)	2 (17)	3 (25)	4 (33)
Fetal abnormality	8 (6)	24.7 ± 7.1	5 (63)	3 (38)	0 (0)	0 (0)
Non-placental IUGR	6 (4)	24.7 ± 2.5	6 (100)	0 (0)	0 (0)	0 (0)
Preeclampsia	4 (3)	29.5 ± 6.0	2 (50)	1 (25)	0 (0)	1 (25)
Abruption	3 (2)	32.9 ± 9.1	1 (33)	0 (0)	0 (0)	2 (67)
Meconium aspiration	4 (3)	39.2 ± 1.5	0 (0)	0 (0)	0 (0)	4 (100)
Explained COD	100 (72)	28.6 ± 7.6	51 (51)	16 (16)	12 (12)	21 (21)
Unexplained COD	38 (28)	28.9 ± 7.5	18 (47)	4 (11)	8 (21)	8 (21)
Overall	138 (100)	28.6 ± 7.6	69 (50)	20 (14)	20 (14)	29 (21)

Values are presented as *n* (%) unless otherwise indicated

*GA* gestational age, *SD* standard deviation, *IUGR* intrauterine growth restriction, *COD* cause of death

**Table 3:** Classification of placental causes of death based on the type of lesion, according to gestational age at autopsy

Type of lesions	Value	GA (mean ± SD)	Extremely preterm	Early preterm	Late preterm	Term
MVM	21 (54)	28.7 ± 5.2	9 (43)	8 (38)	3 (14)	1 (5)
FVM	7 (18)	33.5 ± 5.8	2 (29)	0 (0)	3 (43)	2 (29)
Inflammatory	4 (10)	25.3 ± 11.3	3 (75)	0 (0)	0 (0)	1 (25)
MPVFD	3 (8)	26.1 ± 12.4	2 (67)	0 (0)	0 (0)	1 (33)
DVM	4 (10)	36.0 ± 2.8	0 (0)	1 (25)	1 (25)	2 (50)
Total	39 (28)	29.8 ± 7.0	16 (41)	9 (23)	7 (18)	7 (18)

Values are presented as *n* (%) unless otherwise indicated

*MVM* maternal vascular malperfusion, *FVM* fetal vascular malperfusion, *MPVFD* massive perivillous fibrin deposition, *DVM* delayed villous maturation

Table 4 demonstrate the relevant characteristics and risk factors associated with stillbirth. Unexplained stillbirths had lower rates of underlying maternal medical conditions and bad obstetric history (21% and 24%, respectively). Regarding obstetric complications, cases of ascending infections were frequently seen in the setting of preterm labor and cervical insufficiency (33%). Explained cases of stillbirth were more often small for gestational age (31% vs. 21%) and had abnormal amniotic fluid volume (20% vs. 11%) compared with unexplained cases, while unexplained cases had higher rates of cord lesions of unknown significance (such as single umbilical artery and velamentous cord insertion) (26% vs. 18%). Histopathological findings of unknown significance (isolated increased syncytial knots and low-grade inflammatory lesions) were higher in the explained cases, with highest rates among cases of stillbirth due to cord abnormality and ascending infection (92% and 84%, respectively). When evaluating fetal maceration degree, fetuses of unexplained cases were more macerated compared to the explained cases, a condition that may hamper the pathological examination and impaired the ability to reveal the cause of death (mean maceration degree of 2.7 vs. 2.3, respectively).

**Table 4:** Classification of characteristic and condition associated with fetal death

Cause of death	Value	Maternal history	Abnormal obstetric history	Obstetric complication	SGA	Amniotic abnormality	Mildmalformations	Placental lesions	Cord lesions
Placental lesions	39 (28)	15 (38)	10 (26)	1 (3)	14 (36)	7 (18)	11 (28)	27 (69)	9 (23)
Ascending infection	24 (17)	6 (25)	9 (38)	8 (33)	2 (8)	4 (17)	10 (42)	20 (83)	3 (13)
Cord abnormality	12 (9)	4 (33)	5 (42)	0 (0)	3 (25)	0 (0)	2 (17)	11 (92)	0 (0)
Fetal abnormality	8 (6)	2 (25)	3 (38)	0 (0)	3 (38)	4 (50)	0 (0)	4 (50)	2 (25)
Non-placental IUGR	6 (4)	3 (50)	1 (17)	0 (0)	6 (100)	4 (67)	1 (17)	4 (67)	3 (50)
Preeclampsia	4 (3)	4 (100)	2 (50)	0 (0)	3 (75)	1 (25)	1 (25)	2 (50)	0 (0)
Abruption	3 (2)	1 (33)	0 (0)	1 (33)	0 (0)	0 (0)	0 (0)	2 (67)	0 (0)
Meconium aspiration	3 (2)	0 (0)	2 (67)	0 (0)	0 (0)	0 (0)	1 (33)	2 (67)	1 (33)
Explained COD	100 (72)	35 (35)	32 (32)	10 (10)	31 (31)	20 (20)	26 (26)	72 (72)	18 (18)
Unexplained COD	38 (28)	8 (21)	9 (24)	5 (13)	8 (21)	4 (11)	8 (21)	22 (58)	10 (26)
Overall	138 (100)	43 (31)	41 (30)	24 (17)	37 (27)	24 (17)	34 (25)	94 (68)	28 (20)

Values are presented as n (%) unless otherwise indicated

*SGA* small for gestational age, *IUGR* intrauterine growth restricted

## Discussion

Our study aimed to objectively define the cause of death in a cohort of 138 cases of IUFD who underwent post-mortem examination in the largest tertiary hospital in Israel over a period of 7 years. The mean gestational age at the time of fetal death was 28.6 weeks and half of the cases were extremely preterm (< 28 weeks) which was in line with previous studies [22, 23].

Due to a lack of uniformity, current metrics differ by the criteria incorporated into their classification systems, as some even neglect placental pathology. We designed detailed up-to-date criteria that incorporate clinical reports, medical history, prenatal imaging, and pathological findings. Our study proposes a refined approach that integrates various nomenclatures found in the literature into a comprehensive classification system aimed to provide an objective and evidence-based tool to classify the causes of stillbirth. In our study, the leading cause of death was attributed to placental pathologies (28%), which were revealed by a post-mortem histopathological examination of the placenta. Studies from high-income countries have reported rates of placental-related stillbirths ranging from 11 to 65% [9]. This variance also emphasizes by the lack of standardization in the current metrics, allowing different placental-related conditions to be included in this category (such as placental abruption, IUGR and preeclampsia). In our study, only cases complying with the described strict definition of placental-related cause of death were included in this category. The rate of unexplained stillbirth cases varies widely in the literature. Compared to previous classification systems that emphasize placental findings, such as TULIP (10.2%) and ReCoDe (13.8%) [24, 25], our study demonstrates a relatively high rate of unexplained cases (28%). This can be explained by our strict criteria for defining the cause of death, as depicted in the methods, resulting in more cases being categorized as unexplained, such as those with only a plausible cause.

The main limitation of our study lies within the difficulty of defining a specific definitive cause of death, as no gold standard diagnosing methodology for stillbirth causes exists, and several overlapping mechanisms can always coexist simultaneously. As previously described, determining the primary cause of death is complex and somewhat dependent on the examiner's focus [26]. Nevertheless, the main strength of our study lies in its methodology, designed to avoid subjectivity, achieve a high level of certainty, and distinguish between causal and non-causal conditions. This is exemplified in the classification challenges of preeclampsia-related stillbirths, a multifactorial disease, with the cause of death depending on various factors. As seen in the literature, preeclampsia can be classified as "maternal preeclampsia", especially in cases of late-onset preeclampsia, often linked to maternal comorbidities, or as a "placental condition," focusing on placental pathology particularly lesions associated with early-onset preeclampsia (e.g., MVM lesions) [27]. Conversely, an emphasis on the outcome of preeclampsia may define the cause of death as "fetal asphyxia". In our study, we consistently regarded the primary severe factor initiating the sequence of events leading to stillbirth as the cause of death. Therefore, in the presence of clinical and pathological findings, the cause of death was determined by the primary factor initiating the process, defined as "preeclampsia." This methodology enables differentiation between cases of placental pathology that lack the clinical manifestation of preeclampsia and those with both clinical and pathological preeclampsia, leading to a more precise classification. Establishing a standard methodological guideline is crucial in the evaluation of stillbirths, as it allows for a rigorous criteria to better distinguish between causal and associated factors. This was evident in cases of intrauterine growth restriction (IUGR), a multifactorial condition, primarily associated with placental origin. Our study aimed to distinguish between normal small-for-gestational-age (SGA) infants with no increased risk and those who were prenatally growth restricted. We classified IUGR as "placental condition" only when related placental pathology was evident. Cases with clinical IUGR (confirmed by a prenatally diagnosed FGR), with no substantial placental pathology, were classified as Non-placental IUGR. This differentiation was based on the understanding that IUGR can result from various etiologies, and classifying all cases as placental could lead to the loss of imperative information, and other etiologies which are not always visible could be disregarded. Additionally, to ensure rigorous distinguishing criteria, mild or focal placental lesions were excluded, focusing solely on high-burden, severe, and extensive lesions which are less common in placentas of normal pregnancies. Lastly, a detailed diagnostic criteria were used for the evaluation of all placental reports, based on the Amsterdam consensus criteria, to ensure a uniform definition.

In conclusion, the pathophysiological mechanisms of stillbirth have yet to be sufficiently described. In our study, the largest category of cause of death was attributed to placental pathology, with MVM lesions being the most common. A rigorous up-to-date criteria that incorporates pathological findings and clinical reports may help objectively classify the cause of death and lower the cases of unexplained fetal death.

## Supplementary Information

Below is the link to the electronic supplementary material.

Supplementary file1 (DOCX 15 kb)

## Data Availability

Available upon request.
