# Supplementary material for: Identifying causes and associated factors of stillbirths using autopsy of the fetus and placenta
Source: Arch Gynecol Obstet. 2024 May 1;311(2):237–44. doi: 10.1007/s00404-024-07522-1 (PMC11890226; doi:10.1007/s00404-024-07522-1)
Supplement: Supplementary file 1 — Supplementary file1 (DOCX 15 kb) [file 404_2024_7522_MOESM1_ESM.docx]

| Table S1 – Classification of histopathological lesions based on the Amsterdam placental workshop group consensus | |
| --- | --- |
| Ascending intrauterine infection | |
| Maternal inflammatory response stage 1 | Acute subchorionitis or chorionitis (neutrophils in the subchorial intervillous space) |
| Maternal inflammatory response stage 2 | Acute chorioamnionitis |
| Maternal inflammatory response stage 3 | Necrotizing chorioamnionitis |
| Fetal inflammatory response stage 1 | Chorionic vasculitis or umbilical phlebitis |
| Fetal inflammatory response stage 2 | Involvement of umbilical vessels |
| Fetal inflammatory response stage 3 | Necrotizing funisitis |
| Fetal Vascular Malperfusion (FVM) | Thrombosis – occlusive and nonocclusive thrombosis of fetal vessels |
|  | Avascular villi |
|  | Intramural fibrin deposition (intimal fibrin cushion) |
|  | Villous stromal-vascular karyorrhexis (Hemorrhagic endovasculitis / vasculopathy) |
|  | Stem vessel obliteration (fibromuscular sclerosis, stem vessel endovasculopaty) |
|  | Vascular ectasia |
| Maternal Vascular Malperfusion (MVM) | Placental hypoplasia – placental weight < 10th centile |
|  | Infarction |
|  | Retroplacental hemorrhage |
|  | Distal villous hypoplasia (DHH) |
|  | Accelerated villous maturation |
|  | Decidual arteriopathy |
| Inflammatory lesions | Villitis of unknown etiology |
|  | Chronic villitis, chronic deciduitis, chronic chorioamnionitis, chronic histochitic intervillositis |
| Delayed villous maturation (DVM) | Delayed villous maturation, villous dysmaturity |
| Massive perivillous fibrin deposition (MPVFD) | Massive perivillous fibrin deposition (MPVFD), maternal floor infarction |
| Placental lesions of unknow significance | Scattered Intervillous thrombi, focal sclerotic villi, isolated increased syncytial knots, chorangiosis, small infracts, mild patchy or low grade villitis, chronic deciduitis, isolated abnormal villi morphology, circumvallate placenta |
